# Editorial: Trajectories in Developmental Disabilities: Infancy–Childhood–Adolescence

**DOI:** 10.3389/fpsyt.2022.893305

**Published:** 2022-04-15

**Authors:** Peter B. Marschik, Luise Poustka, Sven Bölte, Herbert Roeyers, Anders Nordahl-Hansen

**Affiliations:** ^1^Child and Adolescent Psychiatry and Psychotherapy, University Medical Center Göttingen and Leibniz Science Campus Primate Cognition, Göttingen, Germany; ^2^Department of Women's and Children's Health, Center of Neurodevelopmental Disorders (KIND), Centre for Psychiatry Research, Karolinska Institutet and Child and Adolescent Psychiatry, Stockholm Health Care Services, Region Stockholm, Stockholm County Council, Stockholm, Sweden; ^3^Division of Phoniatrics, iDN - Interdisciplinary Developmental Neuroscience, Medical University of Graz, Graz, Austria; ^4^Child and Adolescent Psychiatry, Stockholm Health Care Services, Region Stockholm, Stockholm, Sweden; ^5^Curtin Autism Research Group, Curtin School of Allied Health, Curtin University, Perth, WA, Australia; ^6^Department of Experimental Clinical and Health Psychology, Faculty of Psychology and Educational Sciences, Ghent University, Ghent, Belgium; ^7^Department of Education, ICT and Learning, Østfold University College, Halden, Norway

**Keywords:** interdisciplinary/multidisciplinary, child development, neurodevelopmental disorders, developmental science, trajectories, infancy, childhood, adolescence

Research into infant and child development is, at its heart, an interdisciplinary and integrative science. Recent theoretical and clinical developments as well as technological advances have allowed us to approach ontogeny from different angles and opened new possibilities to measure and understand progress and regression in typical and atypical development [e.g., (1–6)]. Scientific endeavors aiming to (i) anticipate disorder specific trajectories, (ii) decipher developmental pathways, (iii) reliably predict outcomes and (iv) define determinants of health and disease in developmental disabilities or infants at elevated likelihood for atypical development have substantially increased over the past few decades [e.g., (7–11)]. With this Research Topic we aimed to comply with this trend and addressed researchers focusing on the study of “Trajectories in Developmental Disabilities from Infancy through Childhood to Adolescence”. The scope of articles in this compilation reflects the interdisciplinarity in the field of developmental science encompassing basic research, clinical studies, and technological sciences on detection and treatment of individuals with developmental disabilities as well as the impact on the healthcare system.

Various aspects of development in the general population, cohorts at elevated likelihood for atypical neurodevelopment or adverse psychosocial outcome, and late detected developmental disorders are covered in this Research Topic. Van Beek, van der Horst et al.; Van Beek, van de Par et al. report on developmental trajectories in very preterm born infants in an 8-year longitudinal approach. Besides following infants with extremely low birth weight (ELBW) or small for gestational age (SGA), the composition of articles in this issue spans a scope from variability in infants' functional brain networks and its association with behavioral functions (Kelsey et al.) to altered structural and functional brain development in children with intellectual disability [ID; Ma et al.]. Exogenous influences on neurodevelopment were reviewed by Notarbartolo di Villarosa do Amaral et al. studying clinical repercussions of mosquito borne infections (Zika virus). In another section focusing on late detected developmental disorders, Orm et al. report about a 10-year-longitudinal project on cooccurring conditions in autism spectrum (ASD) and attention deficit hyperactivity disorder (ADHD). Two further studies on ASD focused on machine learning approaches outlining interventional effects and the role of intellectual capacities on diagnostic procedures respectively (Blanc et al.; Wolff et al.). Studies on the relation of maternal bonding and social competence in preschoolers (Joas and Möhler), the effect of law reforms on coercive measures for young adults with intellectual and developmental disabilities (Geissler et al.), a digital tool to be used for screening of learning disabilities (Xie et al.), and a review on research trends in Down syndrome (Windsperger and Hoehl) add to the complexity of this selected Research Topic. All included articles truly stress the necessity and highlight the impact of longitudinal studies to better understand developmental constraints and potentials and at the same time inherently pointing to the restrictions, we as scientist face, when it comes to implementing longitudinal research paradigms.

Interdisciplinarity is steadily increasing in both research and clinical environments, but we are longing for new innovative frameworks allowing medical sub-specialties, psychological ones, special education, public health, and related fields to meld. Building new grounds in developmental science to reach a deeper understanding of multiple levels of development (molecular, genetic, neuronal, cognitive, behavioral, environmental, etc.), unravel the complexity of its nature, etiology, and trajectories of disorders, is demanding research to liaise on different levels. Scientists, funders, and stakeholders together need to develop structures aiming at bundling resources for multi-discipline and multi-center approaches. There are already a number of such successful undertakings or initiatives, disorder and method focused [e.g., EU-AIMS, EUNETHYDIS (12–14); or funding programs like Collaborative Research Centers, VW-Change of Course, etc.] but also more general ones, such as the German Centers for Child and Adolescent Health, that should serve as point of origin or blueprint to further advance the study of child development, health, and wellbeing. However, we need to be aware, that true interdisciplinary thinking and the more so the establishment of sustainable research structures needs time and cannot be rushed [cf., (15, 16)]. Building common grounds in a complex interdisciplinary field certainly is not trivial and tools for designing and conducting comparable studies but also for evaluating scientific rigor, eligibility, and success are urgently needed. Crossing borders in research disciplines and funding policies will allow developmental science of today become the developmental science of tomorrow.

## Author Contributions

All authors listed have made a substantial, direct, and intellectual contribution to the work and approved it for publication.

## Funding

This work was supported by Volkswagenstiftung–IDENTIFIED, Rett-Elternhilfe e.V., DFG, SFB 1528, FWF KLI811, and the Leibniz Foundation.

## Conflict of Interest

The authors declare that the research was conducted in the absence of any commercial or financial relationships that could be construed as a potential conflict of interest.

## Publisher's Note

All claims expressed in this article are solely those of the authors and do not necessarily represent those of their affiliated organizations, or those of the publisher, the editors and the reviewers. Any product that may be evaluated in this article, or claim that may be made by its manufacturer, is not guaranteed or endorsed by the publisher.
